# Persistence of *Staphylococcus aureus*: Multiple Metabolic Pathways Impact the Expression of Virulence Factors in Small-Colony Variants (SCVs)

**DOI:** 10.3389/fmicb.2020.01028

**Published:** 2020-05-21

**Authors:** Lorena Tuchscherr, Bettina Löffler, Richard A. Proctor

**Affiliations:** ^1^Institute of Medical Microbiology, Jena University Hospital, Jena, Germany; ^2^Center for Sepsis Control and Care, Jena University Hospital, Jena, Germany; ^3^Departments of Medical Microbiology/Immunology and Medicine, University of Wisconsin Medical School, Madison, WI, United States

**Keywords:** infection, persisters, metabolism, small colony variant (SCV), staphylococcus aureus

## Abstract

*Staphylococcus aureus* is able to survive within host cells by switching its phenotype to the small-colony variant (SCV) phenotype. The emergence of SCVs is associated with the development of persistent infections, which may be both chronic and recurrent. This slow-growing subpopulation of *S. aureus* forms small colonies on solid-medium agar, is induced within host cells, presents a non-homogenous genetic background, has reduced expression of virulence factors and presents a variable phenotype (stable or unstable). While virtually all SCVs isolated from clinical specimens can revert to the parental state with rapid growth, the stable SCVs recovered in clinical specimens have been found to contain specific mutations in metabolic pathways. In contrast, other non-stable SCVs are originated from regulatory mechanisms involving global regulators (e.g., *sigB*, *sarA*, and *agr*) or other non-defined mutations. One major characteristic of SCVs was the observation that SCVs were recovered from five patients with infections that could persist for decades. In these five cases, the SCVs had defects in electron transport. This linked persistent infections with SCVs. The term “persistent infection” is a clinical term wherein bacteria remain in the host for prolonged periods of time, sometimes with recurrent infection, despite apparently active antibiotics. These terms were described *in vitro* where bacteria remain viable in liquid culture medium in the presence of antibiotics. These bacteria are called “persisters”. While SCVs can be persisters in liquid culture, not all persisters are SCVs. One mechanism associated with the metabolically variant SCVs is the reduced production of virulence factors. SCVs have consistently shown reduced levels of RNAIII, a product of the accessory gene regulatory (*agrBDCA*) locus that controls a quorum-sensing system and regulates the expression of a large number of virulence genes. Reduced Agr acitivity is associated with enhanced survival of SCVs within host cells. In this review, we examine the impact of the SCVs with altered metabolic pathways on *agr*, and we draw distinctions with other types of SCVs that emerge within mammalian cells with prolonged infection.

## Introduction

The history of *Staphylococcus aureus* infections parallels the history of bacterial infections in general (Proctor, 2016). With the advent of penicillin therapy for *S. aureus* infections in 1944, a dramatic reduction in mortality was seen. However, by 1949, penicillinase was found to reduce clinical efficacy (Jeffery et al., 1949). Even more perplexing was the presence of prolonged infections despite apparently active antibiotics (Wood et al., 2013). Some of these phenomena were anticipated by the studies of Bigger in 1944 who showed that when staphylococci were exposed to penicillin, a small number of survivors remained viable despite exposure to bactericidal antibiotics (Bigger, 1944), and he designated this subpopulation as “persisters.” Since 1944, persisters have been a very reasonable postulate for antibiotic failures. However, the recovery of a defined group of persisters harvested from clinical cases remained limited until work on clinical staphylococcal small-colony variants (SCVs) became more widespread (Proctor et al., 1995). Data have accumulated over the past three decades, and SCVs are the best characterized subpopulation of bacteria recovered from chronic human infections. These SCVs are often extremely difficult to clear even when combined antimicrobial therapies are employed (Loffler et al., 2014; Tuchscherr et al., 2016; Bui et al., 2017). SCVs are characterized by high capacities to enter and survive within host cells and to evade the immune system. Many SCVs exhibit slow growth, reduced membrane potential, attenuated virulence and decreased activation of hypoxia-inducible factors (Proctor et al., 2006; Tuchscherr et al., 2010a; Kahl et al., 2016). The phenotype of SCVs isolated from clinical samples is often unstable and rapidly reverts to a wild-type phenotype (Proctor et al., 1995, 2006; Tuchscherr et al., 2011; Kahl et al., 2016). Although earlier studies emphasized SCVs with reduced electron transport, only a minority of SCVs obtained clinically carry these mutations (Kahl et al., 2016). Further studies revealed SCVs formed by regulatory mechanisms that have been named “dynamic SCVs” (Tuchscherr et al., 2015). As *S. aureus* exploits host cells using them as an intracellular shelter, later adaptations occur and intracellular *S. aureus* form permanent (stable) SCVs (Lattar et al., 2009). These adaptations are discussed in detail in this manuscript. A common characteristic in both SCVs that arise from altered electron transport and regulatory pathway changes is the reduced Agr activity. SCV phenotypes, associated with chronic infections, express fewer virulence factors than wild-type phenotypes and hide within human cells (Proctor et al., 2006; Tuchscherr et al., 2010b). These effects are dependent upon the reduced activity of the Agr system. In this review, an exploration of the pathways that contribute to altered *agr* regulation in stable and non-stable SCVs of *S. aureus* is presented.

## SCVs Versus Persisters

### Definition of SCVs

**FIGURE 1:**
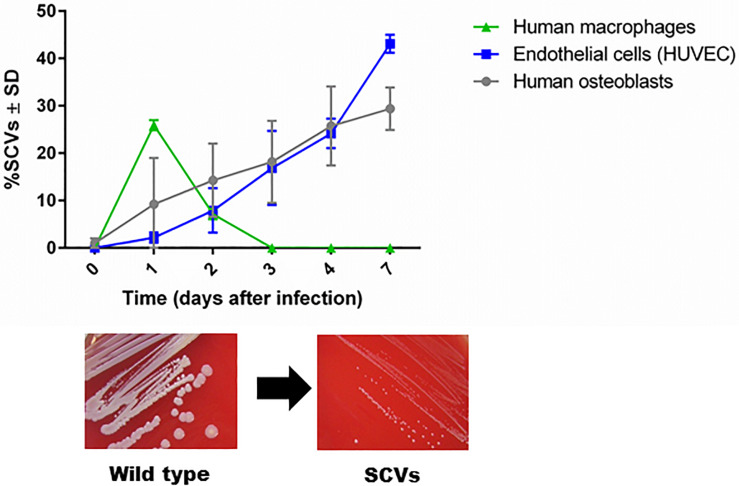
SCV formation in phagocytic and non-phagocytic cells. *S. aureus* survives in higher numbers in non-phagocytic cells, such as osteoblasts and endothelial cells, than in phagocytic cells, such as macrophages. Because macrophages are professional phagocytes, they are able to eliminate intracellular bacteria very efficiently after 3 days, and no remaining bacteria are present to become SCVs. However, enhanced survival of *S. aureus* in osteoblasts and endothelial cells has been observed for up to 7 days. During the intracellular life stage of *S. aureus*, several bacterial cells switch to the SCV phenotype to resist intracellular stress conditions (unpublished data from L. Tuchscherr).

The first description of SCVs dates back more than a century, when they were defined as a subpopulation that grew slowly, producing colonies one-tenth the size of the parent colony or smaller (Proctor et al., 2006). The phenotypic characteristics of SCVs are the formation of small colonies on agar, reduced pigment production, decreased hemolysin production, reduced mannitol fermentation, and a decreased membrane potential, which cause increased resistance to cationic antimicrobials (aminoglycosides, calcium-loaded daptomycin, and cationic antimicrobial peptides) (Proctor et al., 2006). In 1995, chronic infection was associated with the isolation of SCVs with defects in respiration and antibiotic resistance (Proctor et al., 1995). In 2011, dynamic SCVs were defined as a phenotypic subpopulation that appears during the intracellular life stage of *S. aureus* but can rapidly revert back to the original wild-type phenotype via regulatory mechanisms that enable the bacteria to react to changing environmental conditions. Although not all dynamic SCVs present the auxotrophy for menadione, thymidine or hemin, they exhibit all the phenotypic attributes of SCVs (Tuchscherr et al., 2011; Proctor, 2019). Furthermore, clinical SCV isolates are often unstable and revert to their wild-type phenotype when cultured in rich bacterial growth medium (Kahl et al., 2005; Proctor et al., 2006; Edwards, 2012). SCVs are able to invade host cells and evade the host response (Tuchscherr et al., 2010a, 2011). The formation of unstable SCVs within host cells is observed in professional and non-professional phagocytic cells. However, the period of SCV survival is longer in non-professional phagocytes such as endothelial cells and osteoblasts (commonly 7 days) than in macrophages, in which SCVs are eliminated after 3 days post infection (Figure 1, unpublished data from L. Tuchscherr). SCVs recovered from these cells are phylogenetically diverse and may be non-stable SCVs (Tuchscherr et al., 2011). Recently, the molecular mechanism underlying the generation of some unstable SCVs was associated with very large chromosomal rearrangements (Cui et al., 2012; Gao et al., 2015; Guerillot et al., 2019).

**FIGURE 2:**
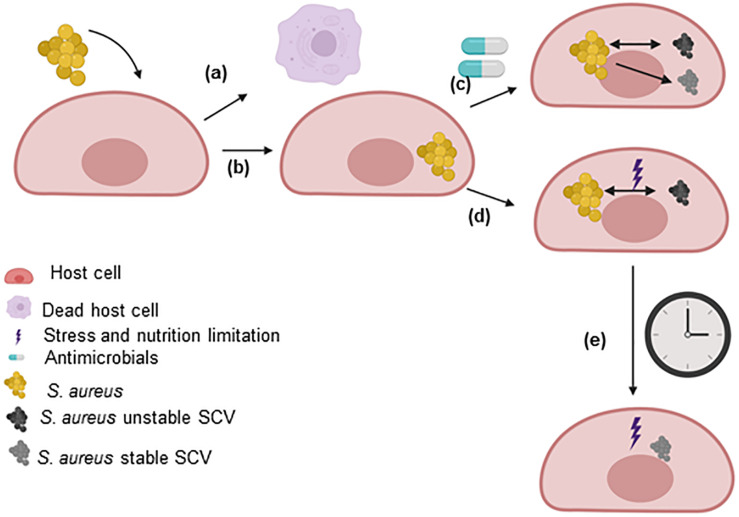
SCV formation within host cells. After the uptake of *S. aureus* by host cells, the two main possibilities are **(a)** the host cell dies (not described in this review) or **(b)** the bacteria survive for extended periods within the host cells. *S. aureus* uses host cells as a shelter to evade the immune response and the effects of antimicrobials. The intracellular environment triggers the selection of bacteria with mutations in metabolic genes. **(c)** However, the utilization of some antimicrobials can select for the formation of persisters that may or may not be SCVs. In this case, the SCVs express a stable or unstable phenotype. **(d)** Within host cells, *S. aureus* has to fight the effects of molecules generated by the host to eliminate the pathogen and the limited nutrient levels (stress). Under these conditions, dynamic SCVs are selected via cross-talk among global regulatory genes. Several SCVs carry specific auxotrophic phenotypes, such as the thymidine-, menadione- and hemin-auxotrophic. This subpopulation can rapidly switch to the original wild-type phenotype in response to environmental conditions (in rich medium, several dynamic SCVs switch to the wild-type phenotype). **(e)** The long-term survival of unstable SCVs of *S. aureus* under stress conditions selects for stable SCVs. This figure was created by Biorender.com.

The development of SCVs has been extensively reviewed (Proctor et al., 1995, 2006, 2014; Proctor, 2019; von Eiff et al., 2006; Tuchscherr et al., 2011; Kahl et al., 2016; Bui et al., 2017). Several pathways have been found to impact the growth rate of *S. aureus* and enhance the formation of SCVs, such as pathways related to energetic supplies (ATP), electron transport, cell wall biosynthesis, global regulatory genes, CO_2_ and fatty acids. These pathways are extensively discussed in this review (section “Pathways involved in the SCV phenotype”). Furthermore, non-stable SCVs may switch to stable SCVs during prolonged survival within host cells, under certain antimicrobial treatments or under certain intracellular stress conditions (Figure 2).

Taking into consideration that SCV features described above, the definition of SCVs should be expanded to include bacterial subpopulation formed within host cells, with variable phenotypic stability, a slow growth rate, decreased expression of virulence factors and alterations in metabolic pathways and/or global regulatory genes. In addition, SCVs can be selected by antimicrobial treatment. In this case, “persister cells” and SCVs are referred to the same definition.

### Definitions of Persisters and Chronic Infection

The term “persister cells” is applied to organisms that remain viable despite active antibiotic exposure. By definition, persister cells represent a slow-growing bacterial subpopulation that tolerates antimicrobial treatment, presents phenotypic variations and is genetically identical to the original bacterial population (Lechner et al., 2012; Bui et al., 2017; Fisher et al., 2017). There are two type of persisters: induced (called type I persistence) and spontaneous (called type II of persistence) (Lechner et al., 2012; Wang et al., 2015; Conlon et al., 2016; Balaban et al., 2019). Induced persisters are generated upon a stress signal, for example starvation. In this case, when the trigger is removed, persister bacteria may maintain their phenotype (Balaban et al., 2019). Spontaneous persiters occur when a fraction of the population switches stochastically to growth arrest during exponential growth (Balaban et al., 2019).

The term “chronic infection” refers to the ability of bacteria to remain viable in the host for an extended period of time. Bacterial survival within host cells takes place when clearance by the host is not sufficient or immune evasion mechanism developed by the pathogen occurs (for example, SCV formation) (Fisher et al., 2017). Persister cells and SCVs have slow growth in common, which reduces the susceptibility to killing by bactericidal antibiotics. Thus, the presence of persister cells as well as SCVs is associated with failure of apparently active antibiotics in clinical practice (Lewis, 2007; Lechner et al., 2012; Garcia et al., 2013; Brauner et al., 2016; Kahl et al., 2016; Vulin et al., 2018; Balaban et al., 2019). Recently, we described that no antimicrobial compounds were effective during chronicity when most bacteria switched into SCVs. Furthermore, the formation of SCVs during infection was enhanced not only by the intracellular environment but also by the actions of certain antimicrobials, such as gentamicin, moxifloxacin and clindamycin, promoting the development of chronic infection (Figure 2; Tuchscherr et al., 2016). Moreover, high levels of antibiotics fail to kill *S. aureus* SCV attached to fibronectin-coated coverslips (Chuard et al., 1997). This review concentrates upon SCVs as a bacterial subpopulation able to avoid the actions of antimicrobials and elimination by the host immune system due to their intracellular location (Proctor et al., 2006; Proctor, 2019; Tuchscherr et al., 2011).

## Clinical Relevance of *S. aureus* SCVs in Chronic Infections

Since 1995, when *S. aureus* SCVs were first linked to chronic and recurrent infections (Proctor et al., 1995), the search for such variants in clinical situations has yielded a large number of studies wherein SCVs cause prolonged infections (Kahl et al., 2016). SCVs have major impacts on the outcomes of infections in the blood, bone, prosthetic joints, brain, skin, pacemakers, and cystic fibrosis lungs (Kipp et al., 2003; Ansari et al., 2015; Kim et al., 2016; Kussmann et al., 2018; Schwerdt et al., 2018; Yang et al., 2018; Loss et al., 2019; Wolter et al., 2019; Wong Fok Lung et al., 2020). Recently, we demonstrated that *S. aureus* strains isolated during nasal colonization, endoprosthesis infection, hematogenous osteomyelitis or sepsis were able to survive within cells and form SCVs (Tuchscherr et al., 2019). These results suggest that staphylococcal strains from different sources are able to develop a chronic infection and form SCVs. Furthermore, the comparison between different strains indicated that low-virulence strains isolated from different sites of infections were able to survive in higher numbers within host cells than were high-virulence isolates (Tuchscherr et al., 2019). Similar results were also found in chronic *in vivo* infection models, such as a hematogenous osteomyelitis model (Tuchscherr et al., 2011, 2015; Tuchscherr and Loffler, 2015) and a chronic mastitis model (Tuchscherr et al., 2008). These results suggest that all staphylococcal strains are potentially able to switch to the SCV phenotype and cause chronic infections associated with treatment failure (Tuchscherr et al., 2016). Several antimicrobials show significant clearance of *S. aureus* during the acute stage of infection; however, all of these antimicrobials fail to eliminate this pathogen during chronicity when the bacterial population is enriched in SCVs. Moreover, treatment of infected cells with low concentrations of gentamicin, moxifloxacin and clindamycin enhances the formation of SCVs (Tuchscherr et al., 2016; Figure 2). The emergence of SCVs during the course of infection indicates a chronic infection in which standard suggested antimicrobial regimens are not sufficient to clear the infection (Bui et al., 2017). Thus, several studies have focused on understanding the formation and metabolism of SCVs to improve the treatment of therapy-refractory staphylococcal infections.

## Pathways Involved in the SCV Phenotype

### Respiration and Virulence Factor Production

The link between respiration and toxin production was noted decades ago when *S. aureus* grown anaerobically on blood agar failed to cause hemolysis. This failure was found to be due to reduced production of α-hemolysin (Hla) (Coleman, 1985). Similarly, the production of toxic shock syndrome toxin-1 (TSST-1) was found to be reduced under strict anaerobic conditions (Sarafian and Morse, 1987). It is now known that the genes for Hla (*hla*) and TSST-1 (*tst*) are regulated by the accessory gene regulatory Agr (*agr*) operon (*agrBDCA*) via RNAIII (Novick et al., 1993; Proctor, 2006). The expression of *rnaIII* is reduced under anaerobic conditions, downregulating Hla and TSST-1 expression (Yarwood et al., 2001). Consequently, a link between respiration and the expression of virulence factors was documented several years ago.

RNAIII is the product of *hld*, which is directly regulated by AgrA, and it is a non-coding regulatory RNA (Novick and Geisinger, 2008). Agr is a quorum-sensing system activated by an autoinducing peptide produced by AgrD and exported by AgrB that activates AgrC to phosphorylate AgrA. AgrA∼P then induces the expression of *hld* to produce RNAIII. The *agr* two-component regulator responds to changes in the environment via multiple regulators (reviewed below).

Respiration and virulence factor production have been studied in detail in staphylococcal SCVs, and defects in electron transport and reduced production of toxins were found (Proctor et al., 2006; Proctor, 2019). While multiple pathways can produce slow growth of *S. aureus*, thereby producing SCVs, respiration-defective SCVs, including thymidine-auxotrophic isolates, consistently show reduced levels of RNAIII (Proctor et al., 2006, 2014).

The associations between respiration and virulence factors might also be observed in dynamic SCVs. However, specific mutations are not always found in this type of SCV (Tuchscherr et al., 2011). Nevertheless, a general characteristic of SCVs is reduced expression of *agr*. Seven days post infection, intracellular bacteria isolated from infected cells and tissues show significant reductions in toxin and *agr* expression (Tuchscherr et al., 2011, 2015; Loffler et al., 2014; Tuchscherr and Loffler, 2015). These results indicate direct effects on bacterial quorum sensing and growth induced by the host intracellular environment, most likely due to nutrient restriction (Vesga et al., 1996; Tuchscherr et al., 2011) and the presence of host cationic peptides (Proctor et al., 2014; Zhang et al., 2018). The key roles of Agr in bacterial adaptation and chronic infections were previously described in several studies (Altman et al., 2018; Suligoy et al., 2018; Sloan et al., 2019). Hence, the downregulation or absence of Agr expression was found to be an enhancer of staphylococcal survival within the host for extended periods (Proctor, 2019).

### Regulation in SCVs

**FIGURE 3:**
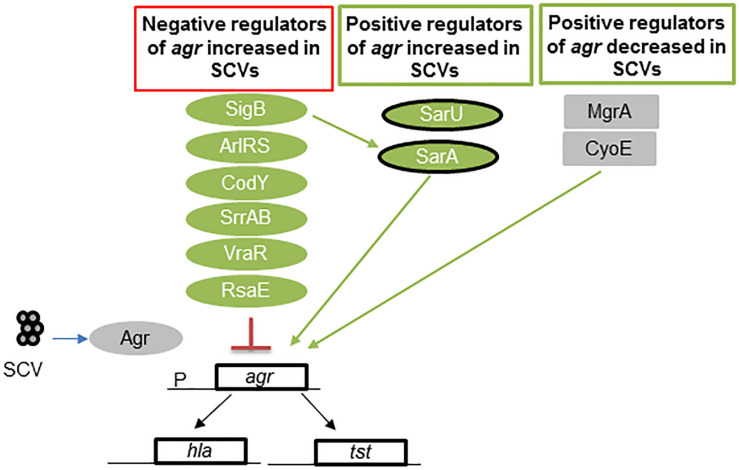
Regulators of *agr* in *S. aureus* SCVs. Low levels of RNAIII, the effector molecule of the Agr quorum-sensing locus, are found in many SCVs. This figure shows the multiple levels of regulation wherein the levels of the positive regulators of *agr* are reduced and those of the negative regulators are increased. Green represents regulators activated in SCVs, and gray indicates no activation in SCVs. SarA and SarU are positive regulators of Agr and exhibit upregulated expression in some SCVs, such as menadione and hemin mutants. However, thymidine-auxotrophic SCVs show decreased SarA levels. Overall, regulation results in a decrease in Agr activation. Abbreviations; sigB: alternative sigma factor B; sarA = staphylococcal accessory regulator; ArlRS = autolysis-related locus two component regulator*;* CodY = GTP-sensing transcriptional pleiotropic repressor; SrrAB = staphylococcal respiratory regulator TCR; VraR = vancomycin resistance-associated regulator of TCR; RsaE = small non-coding RNA E; MgrA = multiple gene regulator A; CyoE = protoheme IX farnesyltransferase; *agr* = accessory gene regulator; *hla* = α-hemolysin gene*;* and *psm* = phenol soluble modulin gene.

In SCVs, negative regulators of Agr exhibit increased expression (SigB, ArlRS, CodY, SrrAB, VraR, and RsaE), and some positive regulators are inhibited (MgrA) or exhibit reduced expression (CyoE and SarA) (Figure 3). These regulators of the Agr operon act to reduce the production of RNAIII (Pragman et al., 2007; Kohler et al., 2008; Pagels et al., 2010; Crooke et al., 2013; Kinkel et al., 2013; Mitchell et al., 2013; Kriegeskorte et al., 2014; Bui and Kidd, 2015; Tuchscherr et al., 2015; Brinsmade, 2017; Stevens et al., 2017; Proctor, 2019). Even though this cross-talk among different regulators has been studied in great detail in respiratory defective SCVs, many of these regulators are also found in dynamic SCVs (Tuchscherr and Loffler, 2015; Tuchscherr et al., 2015, 2017). Thus, several global regulators may be inactivated in other SCVs due to variations in chromosomal structure (Guerillot et al., 2019).

Exceptions to this pattern of negative regulation of Agr, are SarA, and SarU, which were shown to be highly expressed in *hemB* and *menD* mutants and other SCVs (Kohler et al., 2008; Tuchscherr et al., 2015), but they compose a positive regulator of Agr. This is not surprising, as increased SigB and SarA expression is found in SCVs (Senn et al., 2005; Mitchell et al., 2010a, 2013; Crooke et al., 2013; Tuchscherr et al., 2015). However, SarA expression is downregulated in thymidine mutants (Kriegeskorte et al., 2014). Overall, RNAIII production is reduced as a result of the regulatory balance in SCVs, which decreases RNAIII production (Figure 3).

The intracellular survival and formation of SCVs by *S. aureus* in macrophages and in non-phagocytic cells, such as osteoblasts and endothelial cells, was found to be associated with a significant reduction in the Agr level and upregulation of SigB expression (Tuchscherr et al., 2011, 2015). This mechanism of survival and SCV formation involving upregulation of SigB expression was also observed *in vivo* (Tuchscherr and Loffler, 2015; Tuchscherr et al., 2017). SigB increases the expression of adhesins and biofilm-*sarA* genes, resulting in extended survival of *S. aureus* in host cells and patients with cystic fibrosis (Mitchell et al., 2010b; Tuchscherr et al., 2011, 2015). However, it has been shown that S. *aureus* containing simultaneous deletions of *agr*, *sarA*, *and sigB* can survive within host cells for long periods without augmentation of SCV formation (Tuchscherr et al., 2015). These results suggest that *S. aureus* can survive within host cells by modulating the main regulators as found in SCVs, but without the formation of slow growing bacteria. Furthermore, an interesting compensatory mutation, which results in constitutive upregulation of the *srrAB* operon, decreases the growth defects in *men* and *hem* SCV mutants and lead to restored their rapid growth as wild type (Cao et al., 2017). This upregulation does not correct the decreased membrane potential; hence, the increased resistance to aminoglycosides and cationic peptides is maintained (Balwit et al., 1994; Samuelsen et al., 2005). SrrAB is activated by reduced menadione expression via loss of Rex repression (Pagels et al., 2010; Kinkel et al., 2013). Ultimately, SrrAB downregulates RNAIII production, thereby producing a more rapidly growing organism that still carries a number of SCV features (Pragman et al., 2007; Proctor, 2019).

### Impacts of ATP and the Membrane Potential on Prolonged Survival of *S. aureus*

The metabolic pathways involved in the formation of SCVs have very recently been extensively reviewed (Proctor, 2019), so the details of the metabolic changes in menadione- and hemin-auxotrophic SCVs will not be repeated here. However, information on how these respiration-defective variants are involved in bacterial prolonged survival is examined here.

**FIGURE 4:**
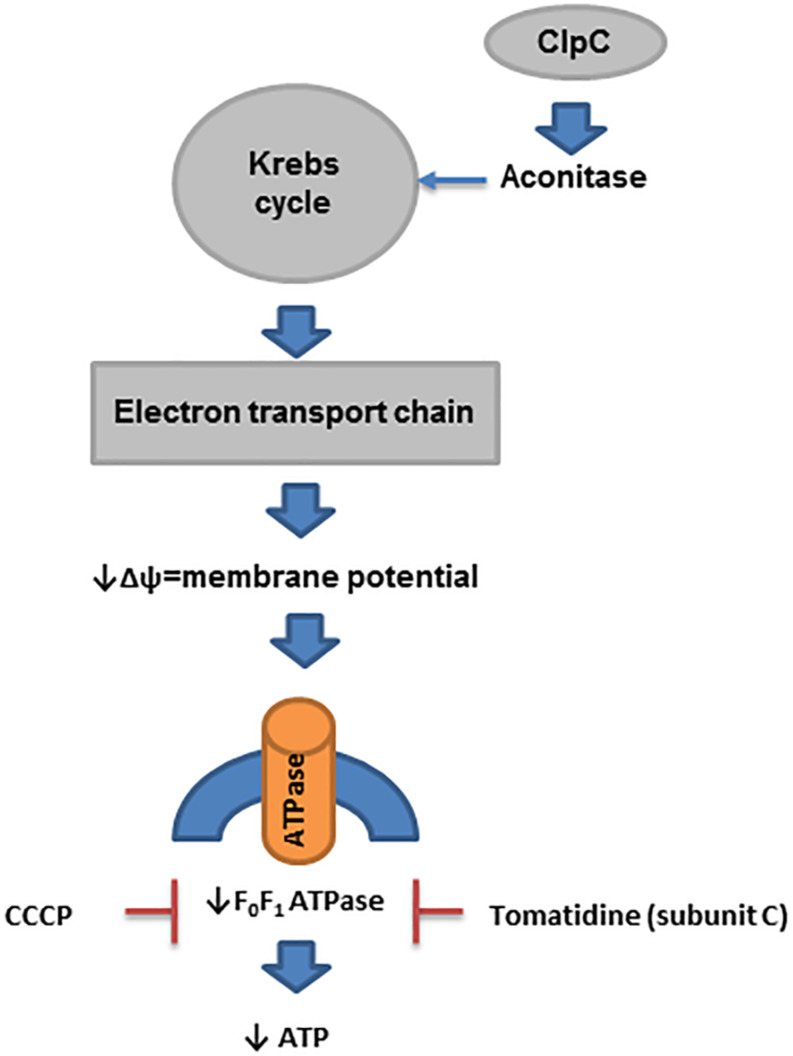
ATP in prolonged survival of *S. aureus*. In SCVs, several pathways that reduce the activity of enzymes in the Krebs cycle have been described. ClpC is needed for the expression of aconitase, and its level is reduced in thymidine-auxotrophic SCVs. Reduced activity of the Krebs cycle decreases the activity of electron transport chain biosynthesis, which produces a reduced membrane potential (Δψ). This process affects the biosynthesis of ATP by F_0_F_1_ ATPase in SCVs.

Many SCVs have reduced electron transport because they have mutations in the genes encoding enzymes used in the biosynthesis of menaquinone and heme (for use in cytochromes) (Proctor et al., 2006). In addition, mutation of *thyA*, which is used in the biosynthesis of thymidine, results in a reduced level of ClpC (caseinolytic protease, a class III group heat shock protein), which is required for the expression of aconitase (Chatterjee et al., 2005, 2007). Reduced aconitase activity decreases Krebs cycle activity, which is linked to the downregulation of electron transport chain biosynthetic enzyme expression (Chatterjee et al., 2007; Wang et al., 2018). Similarly, mutations in the genes for α-ketoglutarate dehydrogenase, *sucA* and *sucB* (enzymes in the Krebs cycle), also result in downregulation of electron transport (Wang et al., 2018). Furthermore, the impact of ClpC on staphylococcal prolonged survival was recently investigated in endothelial cells and keratinocytes (Gunaratnam et al., 2019). Deletion of *clpC* in *S. aureus* enhanced its intracellular survival in both types of cells through modulation of the MazEF system (toxin-antitoxin). However, the formation of SCVs was marginally affected by ClpC (Gunaratnam et al., 2019; Figure 4).

While low ATP levels have been one unifying concept in SCV formation and persister cell formation, more recent data suggest that it is actually the reduced membrane potential that is involved in the prolonged survival of *sucA/B* mutants and not low ATP levels (Wang et al., 2018). Mutations in these genes produce typical SCVs in laboratory strains (Proctor, 2019). Inactivation of *atpA* (ATP synthase) results in no decrease in the ATP level when bacteria are grown in rich medium but does reduce the membrane potential, resulting in a 1000-fold increase in the recovery of persisters (Wang et al., 2018). Of course, reductions in the membrane potential directly impact the formation of ATP via F_0_F_1_ ATPase, which explains why an association between ATP and the membrane potential has been seen. Finally, the proton motive force (PMF) inhibitor carbonyl cyanide m-chlorophenyl-hydrazone (CCCP) also enhances persister cell formation (Grassi et al., 2017; Wang et al., 2018; Figure 4).

Other lines of evidence based on inhibitors of F_0_F_1_ ATPase provide additional support for the concept that reduced PMF is associated with prolonged survival of *S. aureus* within the host. Recent data have shown that F_0_F_1_ ATPase is necessary for SCV survival (Mitchell et al., 2012; Lamontagne Boulet et al., 2018). As implied in its name, F_0_F_1_ ATPase can metabolize ATP and generate a proton motive force when it runs “backward” (Fillingame, 1997). Studies using tomatidine, an inhibitor of ATP synthase subunit C of F_0_F_1_ ATPase and hence ATP production (Lamontagne Boulet et al., 2018), have revealed that this compound is lethal for *men-, hem-*, and *thyA-*mutant *S. aureus* SCVs (Mitchell et al., 2011, 2012). Of note, tomatidine reduces RNAIII production in wild-type *S. aureus* and even in Δ*sigB* strains of *S. aureus* (Mitchell et al., 2012). Another electron transport chain inhibitor produced by *P. aeruginosa*, 4-hydroxy-2-heptylquinoline-*N*-oxid (HQNO) causes normal *S. aureus* to become hyper susceptible to tomatidine (Mitchell et al., 2011). Taken together, these data strongly support the concept that the formation of persisters is related to reductions in the PMF (Figure 4).

Links between ATP levels and chronic infections can still be made when the impact of acyldepsipeptide (ADEP4) on persister cells is examined (Conlon et al., 2013). ADEP4 acts on ClpP, a protease that requires ATP for activation (Kirstein et al., 2009; Frees et al., 2014). Normally, peptides are delivered to ClpP by ATP-dependent ClpX, ClpC, or ClpA subunits. In the presence of ADEP, proteolysis by ClpP no longer depends on ATP. ADEP4 binds to ClpP and keeps the catalytic chamber open, allowing access to peptides and proteins that normally are too large to access the chamber independent of ATP (Kirstein et al., 2009; Conlon et al., 2013, 2016; Frees et al., 2014). Thus, ADEP4 is effective to kill persister cells.

Another link between ATP and persisters was found when it was discovered that metabolic activity, not the growth rate, was critical for antibiotic lethality (Lopatkin et al., 2019). In these studies, ATP was used as a “metabolic reporter” for metabolic activity. Bacterial survival has been inversely correlated with ATP levels in many bacterial species, including *S. aureus.* Metabolically active, but non-dividing, bacteria were found to be more susceptible to antibiotics than dividing bacteria (Yamaguchi et al., 2003; Wang et al., 2018; Pu et al., 2019). Of course, in these studies, ATP levels were measured as the link with persisters.

A final link between ATP and persistence can be found in SCVs. Under aerobic conditions, the production of ATP by F_0_F_1_ ATPase uses oxygen. The use of oxygen by intracellular bacteria activates hypoxia-inducible factor (HIF) within host cells (Taylor and Colgan, 2017; Proctor, 2019). The activation of HIF-1 stimulates the host immune system to remove infected host cells. Respiration-defective *S. aureus* SCVs use less oxygen than wild-type *S. aureus* and fail to activate HIF-1 (Werth et al., 2010), thereby allowing the SCVs to survive within the host.

#### Metabolic Pathways and Mutations Found in SCVs With Normal ATP Levels

While many links can be made between SCVs and ATP, there are several other pathways that can lead to SCV development when ATP levels are not reduced by interruption of electron transport. These SCVs also cause chronic infections and have low RNAIII levels. For example, *S. aureus* with mutations in lipid biosynthesis genes are SCVs. The exposure of *S. aureus* to daptomycin selects for mutations in *fabF* (a fatty acid synthase), which results in an SCV phenotype (Lin et al., 2016). Of interest, *fabF* mutants are auxotrophic for Tween 80, and administration of Tween 80 restores full growth of this mutant. Another clinical *S. aureus* SCV was reported to be auxotrophic for unsaturated fatty acids (Kaplan and Dye, 1976). Recently, some fatty acid-auxotrophic staphylococcal SCVs were found to carry a mutation in the ECF (energy-coupling factor) transporter, which is involved in fatty acid import (Schleimer et al., 2018). These organisms showed reduced hemolysis and decreased pigmentation and formed small colonies. Increased expression of *fabI* also makes organisms triclosan resistant; however, these mutants are more susceptible to other antibiotics (Seaman et al., 2007; Forbes et al., 2015; Bazaid et al., 2018). Other mutations in fatty acid biosynthesis genes, such as *fakAB* (fatty acid kinase), *plsX* (glycerol-3-phosphate acyltransferase), and *accD* (acetyl-CoA carboxylase carboxyl transferase), can also result in fatty acid-auxotrophic SCVs (Parsons and Rock, 2011; Parsons et al., 2013, 2014), but these mutants have not yet been reported in clinical isolates. However, an *Enterococcus faecalis* SCV, which is an unsaturated fatty acid auxotroph, was isolated from a child with chronic omphalitis, but the mutation was not reported (Kubota et al., 2013).

Recently, it was shown that CO_2_ auxotrophs grow as SCVs and have been associated with chronic infections. Non-hemolytic, non-pigmented SCVs that slowly became catalase positive and easily reverted were recovered from 14 patients with chronic infections (Gomez-Gonzalez et al., 2010). In another case report, a chronic breast infection was caused by a CO_2_-dependent *S. aureus* SCV (Bhattacharyya et al., 2015). The activity and levels of *agr*/*rnaIII* were not reported.

Staphylococcal strains that carry a *fusE* mutation grow as SCVs. These strains can be selected by exposure to aminoglycosides and are resistant to fusidic acid (Norstrom et al., 2007; Lannergard et al., 2011). These organisms contain mutations in *rplF* and associated mutations in the *hem* and/or *men* genes. Chronic bacteremia has been caused by antibiotic-resistant *S. aureus* SCV carrying *fus* mutations (Lannergard et al., 2009).

A mutation in cold shock protein B (*cspB*) produces an SCV with decreased pigmentation and resistance to aminoglycosides and trimethoprim-sulfamethoxazole but increased susceptibility to daptomycin, teicoplanin, and methicillin (Duval et al., 2010). As *hemB* mutants show reduced expression of CspB (Seggewiss et al., 2006; Kriegeskorte et al., 2014), it is possible that this decreased expression might be one of the mechanisms underlying prolonged survival of *S. aureus*.

## Conclusion

*S. aureus* is able to respond very rapidly to external stimuli. As soon as this pathogen gains access to the intracellular environment, regulatory cross-talk takes place, and dynamic SCVs are formed. Long-term survival within host cells may target specific genes in electron transport and select menadione-, hemin-, and thymidine-auxotrophic SCVs. The study of these mutants provides information on the pathways involved in the formation of SCV phenotypes that may also be active in dynamic SCVs. Nevertheless, defects in growth, which are characteristic of SCVs, can be compensated by other regulators. Thus, persistent cells can also be rapidly growing organisms that exhibit several features of SCVs. *S. aureus* can readily turn off respiration when growing in an anaerobic environment. Of note, mutations in genes encoding components of the electron transport system that downregulate the expression of virulence factors are commonly found in SCVs. However, these mutations are not always observed in dynamic SCVs, indicating that other mechanisms may affect the Agr system. Changes in gene regulation, which result in profound downregulation of *rnaIII* and virulence factor production, were reviewed. Agr seems to be the key component triggering the changes in *S. aureus* need for survival within host cells, evasion of the immune system and resistance to antimicrobials. In conclusion, the main characteristics of *S. aureus* SCV include a reduced membrane potential, low virulence due to alterations in the Agr system through interactions with other molecules, extended survival within host cells, high resistance to specific antimicrobials and efficient evasion of the host immune system. These features contribute to the failure of clinical treatment for chronic staphylococcal infections.

Further investigation is needed to identify pathways involved in the formation of SCVs to improve the treatment of recurrent staphylococcal infections.

## Author Contributions

LT wrote and designed the manuscript and figures. BL contributed to the conception of this work. RP contributed with the writing of the manuscript and design of the figures.

## Conflict of Interest

The authors declare that the research was conducted in the absence of any commercial or financial relationships that could be construed as a potential conflict of interest.
